# Circulating Myeloid Derived Suppressor Cells (MDSC) That Accumulate in Premalignancy Share Phenotypic and Functional Characteristics With MDSC in Cancer

**DOI:** 10.3389/fimmu.2019.01401

**Published:** 2019-06-19

**Authors:** Peiwen Ma, Pamela L. Beatty, John McKolanis, Randal Brand, Robert E. Schoen, Olivera J. Finn

**Affiliations:** ^1^Department of Immunology, University of Pittsburgh School of Medicine, Pittsburgh, PA, United States; ^2^Tsinghua MD Program, Tsinghua University School of Medicine, Beijing, China; ^3^Division of Gastroenterology, Department of Medicine, University of Pittsburgh School of Medicine, Pittsburgh, PA, United States

**Keywords:** colonic adenomas, intraductal papillary mucinous neoplasm (IPMN), tumor antigen mucin 1, immunosurveillance, myeloid derived suppressor cells (MDSC)

## Abstract

Myeloid derived suppressor cells (MDSC) are a heterogeneous population of immature myeloid cells that accumulate in circulation of cancer patients and at tumor sites where they suppress anti-tumor immunity. We previously reported that in a colon cancer prevention trial of a MUC1 vaccine tested in individuals at increased risk for colon cancer, those who did not mount immune response to the vaccine had higher pre-vaccination levels of circulating MDSC compared to those who did. We also reported that individuals with pancreatic premalignancy, Intraductal Papillary Mucinous Neoplasm (IPMN), had increased circulating levels of MDSC that inversely correlated with spontaneous antibody responses against the pancreatic tumor associated antigen MUC1, abnormally expressed on IPMN. Accumulation of MDSC in cancer and their immunosuppressive role had been well established but their presence in premalignancy was unexpected. In this study we compared MDSC in premalignancy with those in cancer with the hypothesis that there might be differences in the composition of various MDSC subpopulations and their immunosuppressive functions due to different lengths of exposure to disease and/or different tissue microenvironments. In cohorts of patients with premalignant polyps, colon cancer, premalignant IPMN, and pancreatic cancer, we confirmed higher levels of MDSC in premalignancy compared to healthy controls, higher levels of MDSC in cancer compared to premalignancy, but no difference in their subpopulation composition or immunosuppressive capacity. We show that levels of MDSC in premalignancy correlate negatively *in vivo* with spontaneous MUC1-specific antibody responses and *in vitro* with polyclonal T cell proliferation and IFN-γ secretion.

## Introduction

Myeloid derived suppressor cells (MDSC) are a heterogeneous population of immature myeloid cells that accumulate in cancer, auto-immunity, and some chronic inflammatory conditions (1, 2). They suppress the function of multiple immune effector cells and in particular T cells through multiple mechanisms. MDSC can be divided into two major subtypes based on their cell surface phenotype and morphology: polymorphonuclear MDSC (PMN-MDSC) and monocytic MDSC (M-MDSC). Additional subtypes have been proposed, such as the early-stage MDSC (E-MDSC) that lack both macrophage and granulocyte markers and are present in some disease settings (3). MDSC have been extensively studied as components of the tumor microenvironment. A clear positive association has been reported between peripheral blood MDSC levels and cancer stage in multiple tumor types including malignant myeloma, colon cancer and pancreatic cancer (4–6). PMN-MDSC are the major immunosuppressive population of MDSC found in cancer patients' blood and at the tumor site (7). M-MDSC, although fewer in number, can have higher T cell suppressive capacity on a per cell basis and are involved in promoting tumor metastasis and serving as biomarkers of tumor prognosis (8, 9).

MDSC expansion and maturation is driven by a complicated signal network in which Prostaglandin E2 (PGE2) plays a critical role. The presence of PGE2 in the environment is essential and sufficient to redirect development of dendritic cells (DC) into fully suppressive MDSCs in a concentration dependent manner (10). Multiple signals that control MDSC expansion also induce PGE2 production creating a positive feedback loop between Cyclooxygenase2 (COX2) and PGE2 in MDSC, leading to increased production of immunosuppressive factors such as Indoleamine 2,3-dioxygenase (IDO), IL-10, IL-4R, Arg-1, and PGE2 itself, all closely related to MDSC suppressive functions (11–15). Furthermore, production of PGE2 by MDSC stimulates the expression of C-X-C chemokine receptor type 4 (CXCR4) and Stromal cell-derived factor 1 (CXCL12) responsiveness, facilitating the migration of MDSC into sites of inflammation and tumor (16).

Although MDSC and their role in the tumor microenvironment have been extensively studied there is still little information on MDSC in early cancer or pre-cancer. With the advent of sophisticated diagnostic methods and increased emphasis on early cancer detection, premalignant lesions are routinely identified, providing research material for study and a new opportunity to better understand the role of MDSC throughout cancer development.

Colon cancer develops along the path of progression from non-advanced adenomas to advanced adenomas to colon cancer (17), accumulating oncogenic mutations along the way (18). In clinical practice, most adenomas are diagnosed by colonoscopy and removed, followed by long term surveillance for adenoma recurrence (19). Both adenomas and colon cancer are characterized by overexpression of the hyperglycosylated tumor forms of the tumor associated antigen MUC1 (20). Similarly, over 15% of pancreatic cancers develop from premalignant cysts in the pancreas known as intraductal papillary mucinous neoplasm (IPMN) that are lined by multiple layers of proliferative ductal epithelial cells overexpressing tumor forms of MUC1. In a previously reported prophylactic vaccine clinical trial (21), we administered the MUC1 vaccine to patients with a history of advanced colonic adenomas who are at increased long-term risk for colon cancer (22). The vaccine elicited strong anti-MUC1 IgG responses in 17 of 39 participants. Compared to those vaccine responders and healthy age-matched controls, significantly higher levels of MDSC were found in the PBMC of non-responders prior to vaccination. This was the first observation of an accumulation of MDSC in premalignancy and their apparent negative effect on the immune response. We made the same observation in patients with IPMN (23), showing that in this premalignant disease MDSC can accumulate in the peripheral blood like they do in colon pre-cancer.

We questioned whether MDSC in patients with premalignancy would be the same in the composition of phenotypically defined subpopulations and in their immunosuppressive capacity as MDSCs in cancer patients. We prospectively collected PBMC from two cohorts of patients: Colon Cohort, those diagnosed with premalignant or malignant disease of the colon (colon adenoma vs. colon cancer), and Pancreas Cohort, those diagnosed with premalignant IPMN or pancreatic cancer. In both cohorts, PBMC from patients who were screened and diagnosed as healthy (no adenoma, IPMN, or cancer) served as controls. We examined levels of total MDSC and then separately three MDSC subpopulations, monocytic (M-MDSC), granulocytic (PMN-MDSC) and early (E-MDSC) (4). In both cohorts we saw an increase in the percent of total MDSC and the various subpopulations in premalignancy and in cancer compared to healthy controls, with the levels in cancer being generally higher than in premalignancy. There was no difference in the MDSC subpopulation composition. Like in cancer, MDSC isolated from premalignancy directly suppressed *in vitro* T cell proliferation and IFN-γ production. Indirect evidence of their *in vivo* suppressive activity was reflected in decreased levels of spontaneous anti-MUC1 IgG and increased levels in plasma of PGE2 and its metabolite.

## Materials and Methods

### Patients and Sample Collection

For the Colon Cohort, after informed consent (IRB#0411047), blood samples for patients undergoing colonoscopy or colon surgery were obtained prior to onset of the procedure, along with an epidemiologic questionnaire, and permission to access medical records. Specimens were processed under standard operating procedures of the Pittsburgh Biospecimen Core. The collection was supported by a grant from the Early Detection Research Network (UO1CA152753).

For the Pancreas Cohort, samples were obtained as part of the The Pancreatic Adenocarcinoma Gene Environment Risk (PAGER) Study—a prospective cohort study of patients at risk or having pancreatic disease (IRB# PRO07030072). PAGER serves as the universal study for enrolling pancreatic cancer cases and diseased controls subjects at the University of Pittsburgh Medical Center (UPMC) by all of the different medical and surgical disciplines involved in the care of benign and malignant pancreatic diseases. It allows for the collection of biospecimens following the standard operating procedures of the Early Detection Research Network (EDRN) along with associated clinical data including a patient questionnaire and access to the subject's clinical records. Blood samples used in this study were collected on patients prior to any treatment including chemotherapy or surgery.

### Blood Processing, Plasma, and Live PBMC Preservation

Whole heparinized blood was layered on lymphocyte separation medium (MPbio) and centrifuged at 800 g for 10 min with lowest acceleration and deceleration speed, the same day it was drawn. Plasma was collected of the top of the separation tube and frozen in small aliquots at −20°C. PBMC were collected from the interphase between plasma and separation medium, washed once, resuspended in 80% human serum and 20% DMSO and stored in liquid nitrogen.

### MDSC Phenotyping

Previously frozen PBMC were thawed in the 37°C water bath, washed, and stained for Fluorescence Activated Cell Sorter (FACS) analysis with APC labeled anti-human CD11b (BD Biosciences Clone:ICRF44), PE-Texas/Red labeled anti-human CD33 (BD Biosciences Clone:WM53), FITC labeled anti-human HLA-DR (BD Biosciences Clone:G46-6), V450 labeled Anti-human CD14 (BD Biosciences Clone:MϕP9) and PE-Cy7 labeled anti human CD15 (BD Biosciences Clone:HI98). Stained cells were analyzed on IMM Fortessa (BD Bioscience) and data analyzed using FlowJo (v10) software (FlowJo LLC) (21).

MDSC subpopulation phenotypes were defined according to Bronte et al. (4) as follows:

Total MDSC: CD11b^+^HLA-DR^−/low^, CD33^+^
PMN-MDSC: CD11b^+^HLA-DR^−/low^ CD33^+^ CD15^+^ CD14^−^
M-MDSC: CD11b^+^HLA-DR^−/low^ CD33^+^ CD15^−^ CD14^+^
E-MDSC: CD11b^+^HLA-DR^−/low^ CD33^+^ CD14^−^ CD15^−^

### Anti-MUC1 IgG and IFN-γ Enzyme Linked Immunosorbent Assays (ELISA)

For anti-MUC1 IgG, 96-well microtiter plates (Immulon 4, Thermo-Fisher Scientific, MA) were coated with 1 μg MUC1 100mer peptide (the sequence PDTRPAPGSTAPPAHGVTSAx5 corresponding to five 20aa tandem repeats) in 50 μl Delbecco's PBS (DPBS) per well at 4°C overnight. The plate was then washed 3 times with DPBS and 2.5% bovine serum albumin (BSA) was added in 100 μl DPBS for 1 h at room temperature (RT) as a blocking reagent. The plate was emptied, 50 μl of plasma added at 1:40 dilution and incubated for 1 h at room temperature (RT) on a shaker. The plate was then washed five times with 0.1% tween20 detergent in DPBS. Fifty microliter alkaline phosphatase conjugated with anti-human IgG (Sigma-Aldrich) in 2.5% BSA DPBS was added and the plate incubated for 1 h at RT. The plate was washed again, 100 μl of p-nitrophenyl phosphate (Sigma-Aldrich) added and the plate incubated for 1 h in the dark. The reaction was stopped with 50 μl 0.5 M NaOH. The plates were read at OD 405 nm on the spectrophotometer. Control (no antigen) plate was put through the same reactions except that 50 μl DPBS were added instead of the MUC1 peptide. OD values from the no antigen wells were subtracted from corresponding values on the antigen-coated wells. All samples were tested in triplicates.

IFN-γ ELISA was done according to the manufacturer's protocol (Biolegend, Human IFN-r ELISA sets) with cell supernatants from the cultured T cells added at 1:300 dilution.

### Depletion of CD15^+^ Cells From PBMC and T Cell Proliferation Assays

PBMC were isolated from fresh blood and resuspended in 80 μl of MACs buffer [PBS with 0.5% bovine serum albumin (BSA) and 2 mM EDTA]. The cells were then mixed with 20 μl of CD15 MicroBeads (Miltenyi) and incubated at 4°C for 15 min (volume/per 10^7^ total cells). Cells were washed and resuspended in 500 μl of MACs buffer. The cell suspension was applied onto the LS magnetic column (Miltenyi), rinsed by MACs buffer beforehand. The column was washed three times with MACs buffer and unlabeled cells that passed through were collected as CD15^+^ cells-depleted PBMC. T cells were isolated using human Pan T cell isolation beads (Miltenyi) following the manufacturer's protocol with the exception of collecting labeled T cells attached to the column and discarding the unlabeled cells that passed through. Cell purity was analyzed by flow cytometry.

CD15^+^ cells-depleted PBMC or whole PBMC were resuspended at a final concentration of 20 ×10^6^/ml in equal amounts of PBS and Cell Trace Yellow (Thermo-Fisher) at a 1:500 dilution, incubated in a 37°C water bath for 8 min and quenched with pre-warmed PBS for another 8 min. The labeled cells were then resuspended in RPMI 1640 medium supplemented with 10% human serum, 0.5% Penicillin-Streptomycin, 0.5% l-glutamine, 0.5% NEAA, 0.5% pyruvate, 200 IU/ml recombinant human IL-2, with TransAct (Miltenyi) in the experiment group, plated in 96-well round bottom plate and placed in the incubator for 4 days at 5% CO_2_ and 37°C. T cells in PBS with TransAct were used as a positive control and T cells in medium alone as a negative control. On day 4, cells were harvested and culture supernatants collected for IFN-γ ELISA as described above.

To measure proliferation, cells were suspend in 50 μl FACs buffer with added human Fc receptor blocker (BD Bioscience) at ratio of 1:50, incubated on ice for 20 min and centrifuged at 1,400 rpm for 5 min. Cells were then resuspend with 50 μl mixed antibody solution at 1:50 dilution of anti-CD3-FITC (BD Bioscience) and 1:50 dilution of Ghost (TonBo Bioscience) in FACS buffer and stained for 1 h on ice in the dark. Cells were then washed and resuspended in 0.3 ml FACS buffer and analyzed on IMM Fortessa (BD Bioscience). Gating and analysis were done on software FlowJo v10 (FlowJo LLC). Live T cells were gated as Ghost^−^ CD3^+^ and proliferation was shown by Cell Trace Yellow.

### Statistical Analysis

Statistical analyses were performed on GraphPad Prism6 using one-way ANOVA and Student's *t*-test. *P* < 0.05 was considered indicative of statistical significance.

## Results

### Elevated MDSC Levels in PBMC of Patients With Premalignant Colon Adenomas and Premalignant Pancreatic IPMN

**Figure 1:**
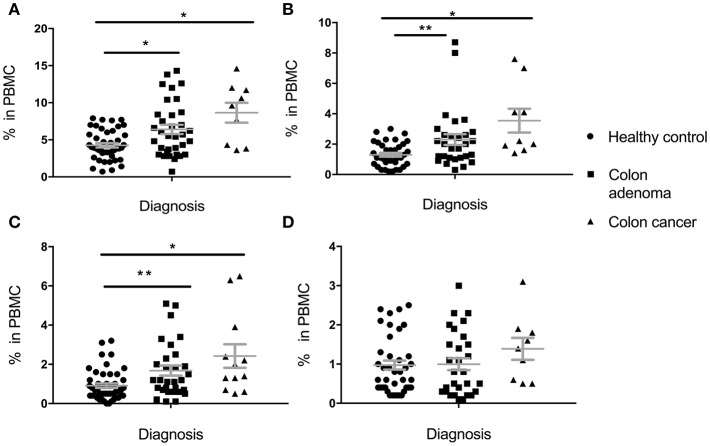
Levels of circulating total MDSC and various MDSC subpopulations in PBMC of individuals diagnosed with adenomas, compared to healthy individuals and individuals with colon cancer. **(A)** Total MDSC; **(B)** PMN-MDSC; **(C)** M-MDSC; **(D)** E-MDSC. Each symbol represents a single individual. Mean with SEM bar for each group is shown in grey. ^*^p < 0.05, ^**^p < 0.01.

Percentage of total MDSC, PMN-MDSC, M-MDSC, and E-MDSC in PBMC was determined based on cell surface marker expression (see section Materials and Methods). In the Colon Cohort (Figure 1), percentages of Total MDSC (Figure 1A), PMN-MDSC (Figure 1B) and M-MDSC (Figure 1C) in premalignant adenoma patients were significantly higher than in healthy controls with all subpopulations still higher in cancer compared to healthy controls. Cells with E-MDSC phenotype (Figure 1D) followed a different pattern. They were present at low, normal levels in healthy controls and patients with adenomas. Their percentages appear to increase in colon cancer, albeit not significantly. There was a trend toward slightly higher levels in cancer than in premalignant samples but it did not reach statistical significance (total MDSC: *p* = 0.1124, PMN-MDSC: *p* = 0.1171, M-MDSC: *p* = 0.1849, e-MDSC: *p* = 0.2207).

**Figure 2:**
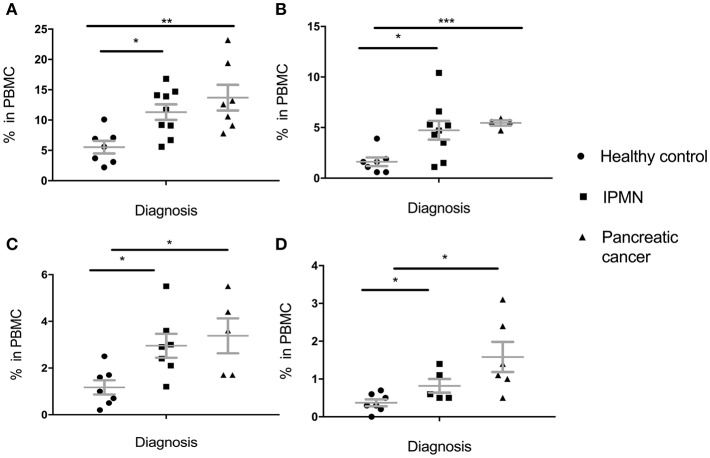
Levels of circulating total MDSC and various MDSC subpopulations in PBMC of individuals diagnosed with premalignant pancreatic intraductal papillary mucinous neoplasms (IPMN) compared to healthy individuals and individuals with pancreatic cancer. **(A)** Total MDSC; **(B)** PMN-MDSC; **(C)** M-MDSC; **(D)** E-MDSC. Each symbol represent a single individual. Mean with SEM bar for each group is shown in grey. ^*^p < 0.05, ^**^p < 0.01,^***^p < 0.001.

These results were in great part replicated in the pancreatic cohort (Figure 2). Percent of total MDSC was higher in IPMN than in healthy controls and even higher in pancreatic cancer PBMC (Figure 2A). This held for all subpopulations, PMN-MDSC (Figure 2B), M-MDSC (Figure 2C) and E-MDSC (Figure 2D). Here again, even though there was a trend toward higher levels in cancer vs. premalignant samples, this was not statistically significant (total MDSC: *p* = 0.3303, PMN-MDSC: *p* = 0.6387, M-MDSC: *p* = 0.6262, E-MDSC: *p* = 0.1386).

### *In vivo* Suppressive Function of MDSC in Premalignancy

**Figure 3:**
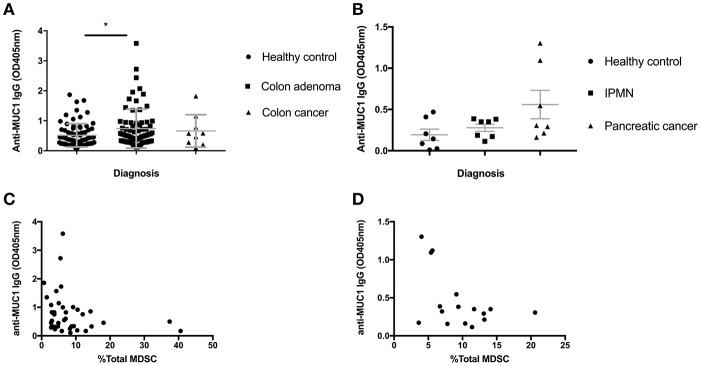
Serum anti-MUC1 IgG levels negatively correlate with MDSC percentages in PBMC in premalignancy. **(A)** IgG levels measured by ELISA in healthy individuals, adenomas and colon cancer. **(B)** IgG levels measured by ELISA in healthy individuals, IPMN and pancreatic cancer. Mean +/– SEM indicated in gray. Analysis was performed using Mann-Whitney test. ^*^p < 0.05 **(C)** anti-MUC1 IgG levels in adenomas correlate negatively with the percent of total MDSC in PBMC; **(D)** anti-MUC1 IgG levels in IPMN samples pooled with adenocarcinoma samples show a trend toward negative correlation with the percent of total MDSC in PBMC. Each dot represents a patient; analysis was performed using Spearman correlation.

Colon adenomas and IPMN express abnormal MUC1 found also in colon and pancreatic cancer. We previously published that many patients with those premalignant conditions, similar to many cancer patients, mount a specific anti-MUC1 antibody response (23, 24). As antibodies are known to play a role in tumor immunosurveillance and MDSC are known to suppress most immune effector mechanisms including B cells, we asked if increases in MDSC we described above could have influenced the ability of individuals with premalignancies to mount anti-MUC1 antibody responses. We tested all individuals in the Colon Cohort (Figure 3A) and the Pancreas Cohort (Figure 3B) from whom we had both PBMC and plasma saved, for anti-MUC1 IgG. In the Colon Cohort, the adenoma group had the highest average level of anti-MUC1 IgG. As would be expected from a progressively more immunosuppressive microenvironment, as the disease progressed to colon cancer, fewer individuals in those groups made anti-MUC1 IgG. We then paired the percent MDSC with anti-MUC1 IgG level for each patient with adenoma (Figure 3C). We found that MDSC levels negatively correlated with the anti-MUC1 IgG levels (*p* = 0.0419, *r* = −0.3232).

We had previously published a similar result in patients with IPMN, which we wanted to confirm in this new Pancreas Cohort and to compare with the Colon Cohort. We had a much smaller number of IPMN patients this time so we combined them with the cancer patients, some of whom were positive for anti-MUC1 IgG. We again see that patients with IPMN and cancer show higher average levels of anti-MUC1 IgG compared to healthy donors and importantly when IgG OD of each patient was paired with the same patient's percent of MDSC (Figure 3D), there was a negative correlation (*p* = 0.132, *r* = −0.3941).

**Figure 4:**
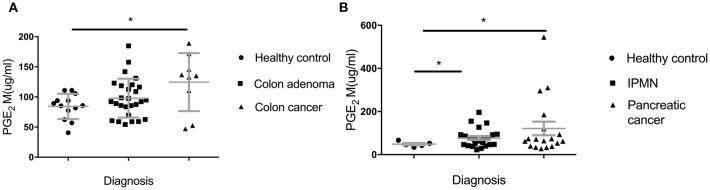
Serum levels of Prostaglandin E2 metabolite (PGE2M). **(A)** Patients with adenomas and colon cancer and healthy controls; **(B)** IPMN and pancreatic cancer compared to healthy controls. PGE_2_M concentration was measured by ELISA. Each dot represents an individual patient. Analysis was performed using unpaired student *t*-test. ^*^p<0.05.

In addition to looking at lower IgG levels as indicators of *in vivo* suppressive effects of MDSC, we sought another biomarker of their presence and *in vivo* suppressive function. PGE2 (Prostaglandin E2) largely contributes to the generation of MDSC from immature myeloid cell and their proliferation and acquisition of inhibitory function (25). We measured PGE2 metabolite in sera of all the groups in the Colon and the Pancreas Cohorts (Figure 4). We found a significant increase of PGE2M in the IPMN group (Figure 4B) (*p* = 0.0136) and a trend toward higher levels in the adenoma group (Figure 4A) compared to healthy controls (*p* = 0.1139).

### *In vitro* Suppressive Function of MDSC From Premalignancy

**Figure 5:**
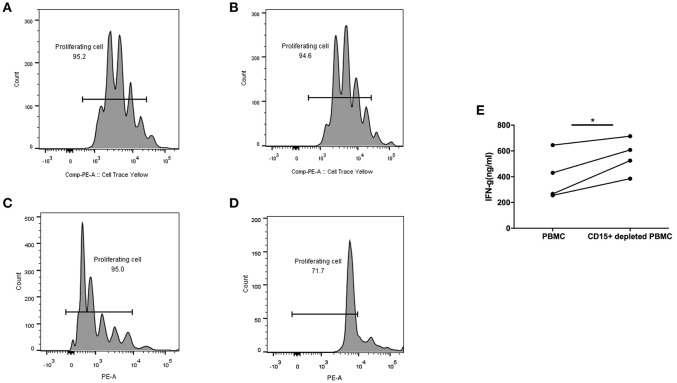
CD15+ MDSC in PBMC from colon adenoma patients suppress their T cell proliferation and Interferon-g production. T cells from Patient 1 **(A,B)** and Patient 2 **(C,D)** in either whole PBMC **(A,C)** or after depletion of CD15+ cells **(B,D)** were activated and their proliferation measured by SFSC dilution 4 days later. **(E)** Interferon-g production by T cells in PBMCs from colon adenoma patients activated in whole PBMC or after depletion of CD15% MDSC, ^*^p < 0.05.

All the above experiments were performed with previously frozen PBMC and plasma. For *in vitro* functional studies of MDSC it was necessary to use fresh blood, which put a limitation on the number of samples we were able to test. We obtained blood from advanced colon adenoma patients one at a time and processed PBMC the same day. We were interested in measuring the function of T cells in each sample in whole blood with MDSC present or after their depletion, which we accomplished by removing CD15^+^ cells, the majority of which are MDSC. T cells in whole PBMC or MDSC-depleted PBMC were activated with Human IL-2/TransAct (Miltenyi) and cultured for 4 days. T cell proliferation and IFN-γ production were measured and compared between the two groups. Figure 5 shows two patients with premalignant colonic adenomas. One had 4% MDSC (CD15^+^) in PBMC (Figures 5A,B) and the other had 31% (Figures 5C,D). In the case of low to normal numbers of MDSC (4%), T cell proliferation rate was the same in whole PBMC (Figure 5A) and after MDSC depletion (Figure 5B). On the other hand, in the setting of high MDSC levels (31%), proliferation of T cells was inhibited in whole PBMC (Figure 5C) but restored after CD15^+^ cell depletion (Figure 5D).

Furthermore, in another 4/4 PBMC samples from colon adenoma patients, activated T cells in CD15—depleted PBMC secreted higher levels of IFN-γ compared to T cells in whole PMBC (*p* < 0.05).

## Discussion

One of the most important findings that came from several decades of basic and preclinical work in tumor immunity and from many failed attempts at immunotherapy, was the highly immunosuppressive nature of the tumor microenvironment, both at the tumor site as well as at a distance, such as in the circulation. We were the first to describe the phenomenon of granulocytes co-sedimenting with white blood cells on a density gradient only in the blood from cancer patients and not from healthy age-matched controls (26). We also showed that numbers of CD15^+^ cells that we characterized initially as granulocytes in the PBMC correlated inversely with patient survival in three different cancers, colon, breast and pancreas. Importantly, we determined that those were activated granulocytes and we were able to recapitulate *in vitro* their capacity to suppress T cells. Those cells are now known as granulocytic MDSC, or PMN-MDSC (CD11b^+^HLA-DR^−/low^ CD33^+^ CD15^+^ CD14^−)^, one of several subpopulations of MDSC responsible for profound suppression of anti-tumor immunity and failure of anti-tumor immunotherapy. The others that we assayed for in this study were subpopulations described in the review by Bronte et al (4), monocytic or M-MDSC (CD11b^+^HLA-DR^−/low^ CD33^+^ CD15^−^ CD14^+)^ and early, or E-MDSC (CD11b^+^HLA-DR^−/low^ CD33^+^ CD14^−^ CD15^−)^. We also looked at the entire heterogeneous population that we referred to as Total MDSC (CD11b^+^HLA-DR^−/low^, CD33^+)^.

MDSC have been reported in several chronic inflammatory diseases (27, 28) but it was only very recently that they were also seen to play immunoinhibitory role in premalignant disease. We first observed their presence in the PBMC of patients with premalignant pancreatic disease, IPMN (29), and later also in patients with premalignant colonic polyps. In the latter, their presence in the PBMC correlated with the inability to respond to a vaccine based on the MUC1 antigen abnormally expressed on colonic polyps and colon cancer, which was being tested for colon cancer prevention (21).

Our observation that MDSC are present in the premalignant as well as the malignant tumor microenvironment begged the question of whether they shared some or all of their phenotypic and functional characteristics. We expected that exposure of MDSC to the premalignant microenvironment would have been of a shorter duration than exposure to the entire process of tumor development and that this would make MDSC in premalignancy in some way different than those described in tumors. While we did not exhaust all the possible comparisons, we can conclude from data obtained in this study that in both premalignant and malignant disease, all phenotypically defined MDSC populations are present and they are immunosuppressive. The only difference appears to be quantitative with the higher numbers generally present in cancer patients. We can also conclude from our *in vitro* T cell experiments that PMN-MDSC are the main immunosuppressive population in these two cancers as depletion of CD15^+^ cells that spares M-MDSC, eliminates most of the suppression of T cell proliferation and interferon production.

As much as we did not see significant differences between MDSC in premalignancy vs. cancer, we conclude that both conditions can lead to their accumulation and their equally immunosuppressive phenotype. Depletion of these cells, which is a goal of several pharmaceutical companies working on potential reagents that could be used for such a purpose, might be considered not only for improving cancer outcome but also for reducing the risk of progression from premalignant disease to cancer. Furthermore, our hypothesis that we might find differences due to among other factors, the length of time that the premalignant lesion has been in the body compared to cancer, might be more applicable to T cells than MDSCs. We showed in our earlier publication (21), and again in this paper, that when removed from the influence of MDSC, T cells in premalignancy regain their normal proliferation and IFN-γ production. This is not the case with T cells from cancer patients that in most cases remain exhausted and dysfunctional (30, 31). Thus, rescuing T cells in premalignancy by removing MDSC or countering their effects in other ways, may give much better results than similar manipulations in cancer.

## Data Availability

All datasets generated for this study are included in the manuscript and/or the supplementary files.

## Ethics Statement

This study was carried out in accordance with the recommendations of the University of Pittsburgh Institutional Review Board with written informed consent from all subjects. All subjects gave written informed consent in accordance with the Declaration of Helsinki. The protocols #0411047 and #PRO07030072 were approved by the University of Pittsburgh IRB.

## Author Contributions

PM performed all the experiments with the help from PB and JM, analyzed results and wrote the first draft of the manuscript. The study was conceived by OF, RS, and RB and supervised by OF. The manuscript was reviewed, revised, and edited by all authors.

### Conflict of Interest Statement

The authors declare that the research was conducted in the absence of any commercial or financial relationships that could be construed as a potential conflict of interest.
